# Contrast enhanced CT on PET/CT imaging in clinical routine: an international survey

**DOI:** 10.3389/fmed.2023.1290956

**Published:** 2023-10-16

**Authors:** Salvatore Annunziata, Nathalie Testart, Katharina Auf der Springe, Marco Cuzzocrea, Marie Nicod Lalonde, Niklaus Schaefer, John O. Prior, Valentina Garibotto, Giorgio Treglia

**Affiliations:** ^1^Unità di Medicina Nucleare, GSTeP Radiopharmacy, Fondazione Policlinico Universitario A. Gemelli IRCCS, Rome, Italy; ^2^Nuclear Medicine and Molecular Imaging Department, Lausanne University Hospital, Lausanne, Switzerland; ^3^Clinic of Nuclear Medicine, Imaging Institute of Southern Switzerland, Ente Ospedaliero Cantonale, Bellinzona, Switzerland; ^4^Faculty of Biology and Medicine, University of Lausanne, Lausanne, Switzerland; ^5^Laboratory of Neuroimaging and Innovative Molecular Tracers (NIMTlab), Geneva University Neurocentre and Faculty of Medicine, University of Geneva, Geneva, Switzerland; ^6^Division of Nuclear Medicine and Molecular Imaging, Geneva University Hospitals, Geneva, Switzerland; ^7^Centre for Biomedical Imaging, University of Geneva, Geneva, Switzerland; ^8^Faculty of Biomedical Sciences, Università della Svizzera Italiana, Lugano, Switzerland

**Keywords:** PET, CT, contrast, enhancement, survey

## Abstract

**Aim:**

To perform an international survey about PET/CT imaging with contrast enhanced CT (PET/ceCT) in clinical routine worldwide.

**Methods:**

A questionnaire of ten questions was prepared for health professionals, addressing the following issues: (1) general demographic, hospital, and department information; (2) use and diffusion of PET/ceCT worldwide; (3) factors influencing the use of PET/ceCT. An invitation to the survey was sent to the corresponding authors of NM scientific articles indexed in SCOPUS in 2022 and dedicated to PET/CT imaging. Data were analysed per individual responder.

**Results:**

191 individual responders worldwide participated in this survey. Most of the responders are using PET/ceCT in their center (74%). Interestingly, the relative use of PET/ceCT over the total PET/CT scans has an anti-Gaussian distribution (<20% ceCT and > 80% ceCT were most represented). Most of responders are using PET/ceCT in oncological settings (62%) and irrespectively from radiopharmaceuticals (62%). In most cases, PET/ceCT scans are reported by NM physicians alone or together by NM physicians and radiologists with an integrated report (31%).

**Conclusion:**

PET/ceCT imaging is largely used worldwide. Local factors can affect the choice of PET/ceCT in respect to conventional PET/CT imaging. Further cost–benefit analysis could be useful to consider other possible influencing variables, such as technologies, dosimetry, department organization and economics.

## Introduction

In the 21st century, Positron Emission Tomography (PET) imaging with 18F-Fluorodeoxyglucose (18F-FDG) and other radiopharmaceuticals emerged as a precious diagnostic tool in several clinical settings, including oncology, neurology, medicine and surgery, thanks to the added functional information in diagnosis, staging, restaging and treatment evaluation of an increasing number of diseases (1).

PET scanners with co-registered Computed Tomography (CT) quickly became the standard technologies (so called hybrid PET/CT), first because of the need of CT for attenuation correction of PET but also offering CT fundamental support for anatomical localization of PET functional findings with several technological improvements in the last years (2), in line with the increasing use of PET/CT worldwide. With the aim of anatomical localization and attenuation correction, co-registered CT scans can have low-dose CT protocols. Nevertheless, hybrid PET/CT scanners are ideally able to perform contrast-enhanced CT (ceCT), adding more precise anatomical and clinical information to the reports. In a cost–benefit evaluation, impact on clinics, dosimetry to patients and department organizations are sensitive points to be evaluated by the Nuclear Medicine and Radiology communities and associations (3).

Therefore, the use of conventional PET/CT and the option for PET/ceCT are still discussed and heterogeneous around the world. Diagnostic and clinical guidelines usually suggest the use of PET/CT and ceCT in the workflows of several diseases, but the choice of co-registrated PET/ceCT often relies on local or individual factors, not yet systematically investigated, or recognized (4).

For these reasons, this international panel of Nuclear Medicine physicians elaborated an international survey addressed to a large community of health professionals, with the aim to investigate the use of PET/ceCT in different departments worldwide, to evaluate specific demographic or hospital characteristics, and to define possible factors influencing the choice of co-registrated PET/ceCT respect to conventional PET/CT in their centers.

## Methods

### Survey preparation

A web-questionnaire was prepared by the authors to evaluate the use of PET/ceCT worldwide, in line with recent recommendations and requirements, to maximize response rates, such as: a personal introductory statement; the offer to make results public; the use of simple headers and textual representation of response categories; the prerequisite of a relatively short deadline including multiple reminders (5).

Ten questions in the English language were prepared for health professionals dedicated to PET/CT imaging worldwide (Appendix A). Question types were dichotomous, single-choice, multiple-choice, rating scales, or open-ended for number, text comment (5). Questions about the use of PET/ceCT worldwide were prepared to address the following main issues: (1) demographic and hospital information; (2) use and diffusion of PET/ceCT worldwide; (3) factors influencing the use of PET/ceCT.

### Survey invitations

The questionnaire was placed in Google Form Document (Appendix A). On the 1st of February 2023, an invitation to the survey was sent to the corresponding authors of scientific articles in the field of PET/CT imaging indexed in SCOPUS in 2022, using the following query string: (“PET”) OR (“positron emission tomography”). Two reminders were mailed to all non-responding recipients. To further increase potential responses, the survey weblink was accessible by electronic devices and social media.

### Data collection and analysis

For this report, the responses received until the 1st of May 2023 were evaluated anonymously. All responses were checked for completeness and collected in a Microsoft Excel table.

**Figure 1:**
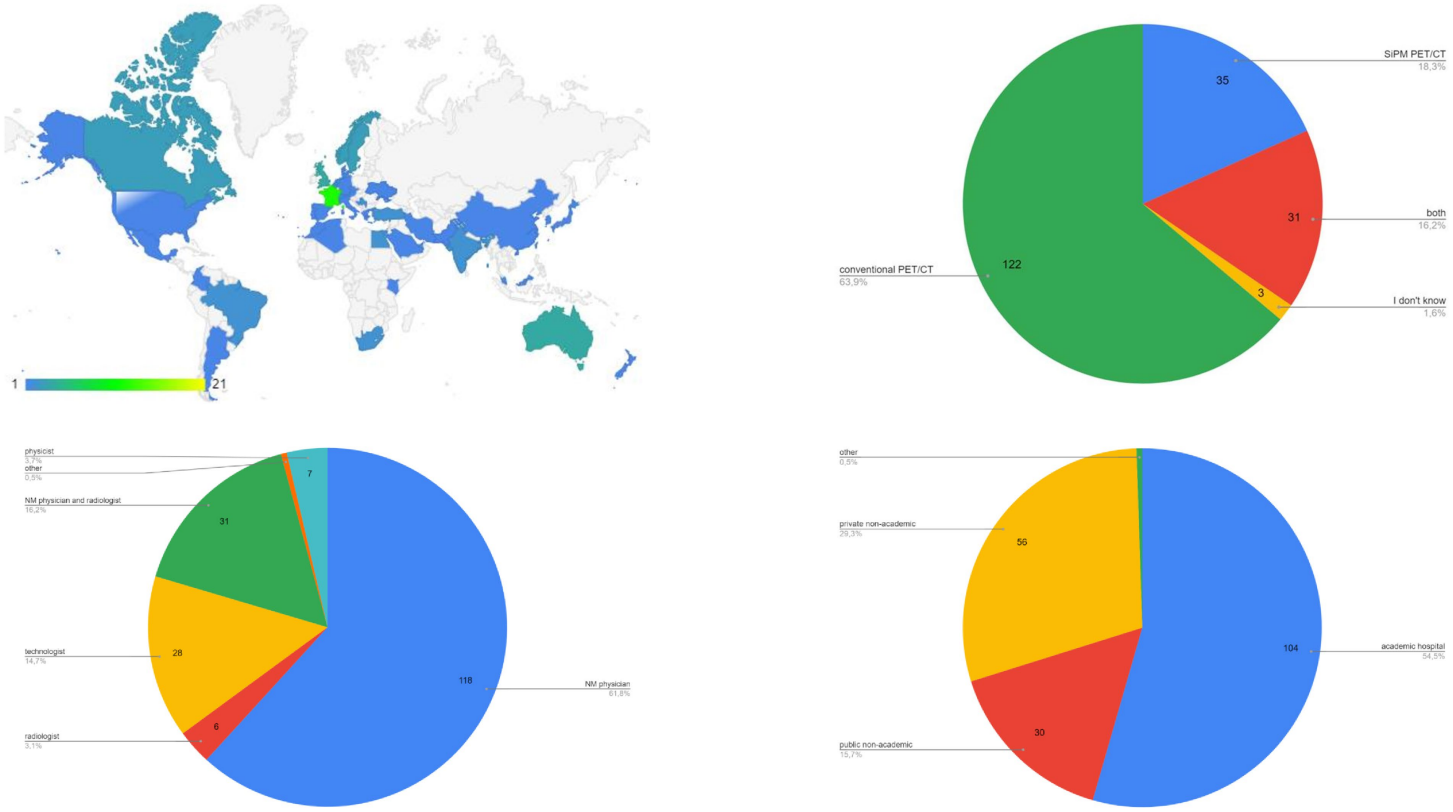
Data about demographics and general information.

**Figure 2:**
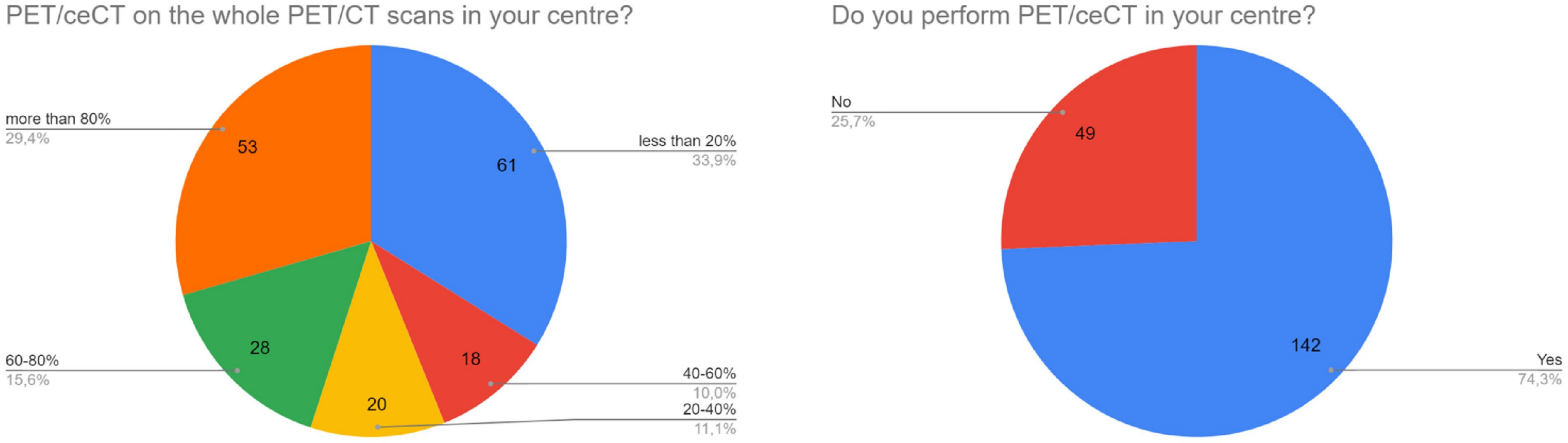
Data about use and diffusion of PET/ceCT worldwide.

**Figure 3:**
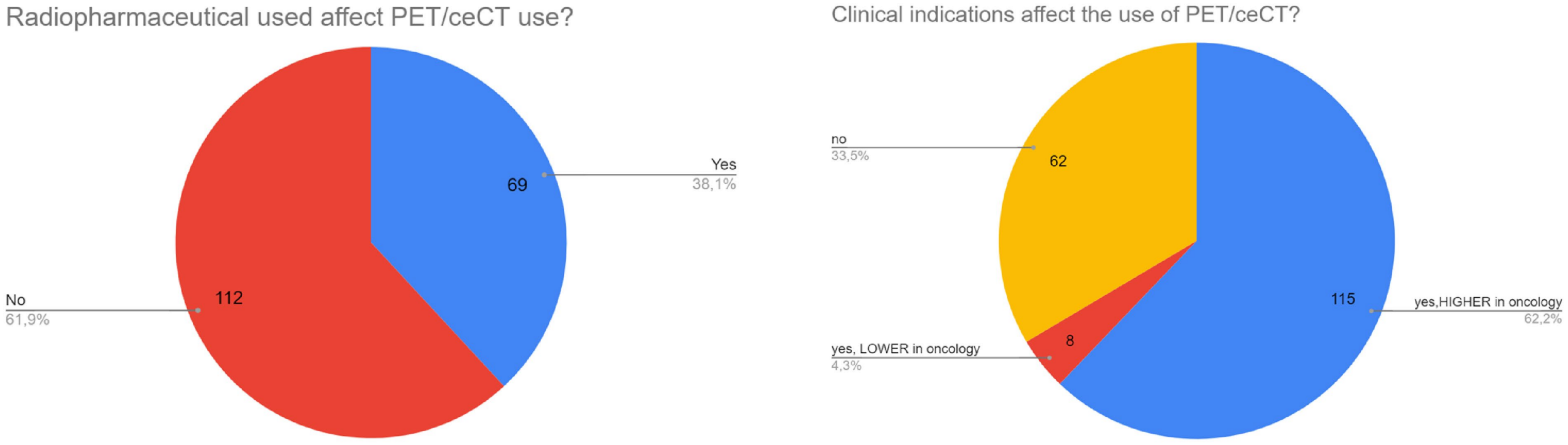
Data about factors influencing the use of PET/ceCT.

All data about demographics, hospital, department, protocols and indications were analysed per individual responder. To perform a report of this survey, we counted the total number of responses per answer option, the proportion of responses per respective answer option in percent, the rank of answer options, or median (range) for a quantitative response (5). Questions about the routinary use of PET/ceCT were optional, so the total number of responders may vary for each questions (Appendix A; Figures 1–3). Free text responses were summarized into categories.

## Results

### Demographic and general information

In total, 191 individual responders participated to the survey. Most represented countries were Italy, Switzerland, France, India and Australia (>10 responders per country, Figure 1). Most of responders were NM physicians (62%, Figure 1), from academic hospitals (54%, Figure 1), using conventional PET/CT scanners (64%, Figure 1), respectively.

### Use and diffusion of PET/ceCT worldwide

A high prevalence of responders is using PET/ceCT in their centers (74%, Figure 2). Interestingly, the relative use of PET/ceCT over the total PET/CT scans has an anti-Gaussian distribution: the most represented situations were “<20% ceCT” and “>80% ceCT” (both around 30%, Figure 2).

### Factors influencing the use of PET/ceCT

Oncological settings can influence the use of PET/ceCT respect to conventional PET/CT, according to the most part of responders (62%, Figure 3). At the same time, the use of a specific radiopharmaceutical seems not to affect the choice of PET/ceCT (62%, Figure 3). Interestingly, 18F-FDG, 68Ga-peptides and PSMA radioligands seem to be the most used tracers for PET/ceCT.

In most cases, PET/ceCT scans are reported by NM physicians alone or together by NM physicians and radiologists with an integrated report (both around 31%).

In the last open question, we asked to write the three most common indications for PET/ceCT in the respective departments, resulting in a wide range of possible diseases and applications, mostly in oncology (lymphoma, lung cancer, abdomino-pelvic cancer, melanoma, NET).

## Discussion

A Nuclear Medicine panel promoted this international survey about PET/CT imaging with contrast enhanced CT (PET/ceCT) in clinical routine worldwide. A questionnaire of ten questions was prepared for health professionals, addressing some issues as general information, diffusion of PET/ceCT imaging worldwide and factors influencing the use of PET/ceCT.

According to this survey, PET/ceCT is largely used, in a variety of countries and in different continents (Figure 1). Nowadays, a large number of available PET/CT scanners offer the possibility to use conventional PET/CT or PET/ceCT worldwide. Interestingly, this choice seems to differentiate between low-users (<20% of PET/ceCT over the total) and high-users (>80% of PET/ceCT over the total, Figure 2). So, local organization and individual preferences probably still affect the use of PET/ceCT. According to a recent editorial, several papers investigated the role of PET/ceCT in different clinical settings (1,670 papers in the timeframe 2010–2021), with a prevalence of articles published in Nuclear Medicine journals, demonstrating a large interest on this topic in scientific literature and clinical routine (6). Available guidelines make suggestions about the use of PET/ceCT in specific clinical scenarios (7), but the real-world dissemination of this tool seems to still rely on subjective factors.

In this survey, oncological settings seem to affect the use of PET/ceCT over conventional PET/CT (Figure 3), but this data should consider the absolute larger use of PET/CT in oncology respect to other non-oncological settings worldwide. A wide variety of possible diseases and applications emerged as most common indication for PET/ceCT among responders, mainly lymphoma, lung cancer, abdomino-pelvic cancer, melanoma, and NET. This is in line with available guidelines suggesting possible added value of PET/ceCT in the diagnostic work-up of these diseases (7). In particular, the use of hybrid PET/ceCT in oncology seems to offer advantages in terms of higher diagnostic accuracy (e.g., oncological staging), time-saving (“one-shot” imaging) and multidisciplinary integration between different clinical professionals (7–10).

Conversely, specific radiopharmaceuticals seem to have no impact on the choice of PET/ceCT, but also in this case the availability of different radiopharmaceuticals other than 18F-FDG should be considered, especially in low-income countries. Interestingly, 18F-FDG, 68Ga-peptides and PSMA radioligands seem to be the most used tracers for PET/ceCT. This is line with recent literature, suggesting possible role of PET/ceCT in NET and prostate cancer management (1, 8, 9).

Moreover, other technical and administrative points could be considered about the use of PET/ceCT. Consistent guidelines do not exist for the acquisition of PET/ceCT, and CT protocols used in PET/CT are not supported by a robust scientific literature regarding acquisition parameters, IV contrast administration, and their contribution to dosimetry (6, 7). Ideally, higher costs, integrative NM/radiology workflows and dose to patients due to ceCT seem to negatively affect the use of PET/ceCT in routine. Nevertheless, the possibility of a one-shot imaging, “fully-hybrid” reports and new low-dose PET/ceCT algorithms could play a role in favour of PET/ceCT in respect to conventional PET/CT imaging (6, 7, 10). Furthermore, in our survey we could not estimate in how many patients an additional ceCT would have been performed or prescribed in case of a conventional PET/CT investigation. Further cost–benefit analyses could be useful to consider all these influencing variables.

The present study has some limitations. Even though we used systematic criteria for survey invitation and dissemination, some potential responders might not have been reached or did not answer to the questionnaire. In particular, the survey was completed by a limited number of subjects and most of them came from Europe. Data analysis was done according to individual responders, to introduce no selection bias in the responses. Some confounding factors emerged in the cohort such as on demographics, but we proposed possible evaluations and solutions in this paper.

## Conclusion

PET/ceCT imaging is largely used worldwide. Local factors can affect the choice of PET/ceCT in respect to conventional PET/CT imaging. Further cost–benefit analysis could be useful to take into account several possible influencing variables, such as technologies, dosimetry, department organization and economics.

## Data availability statement

The raw data supporting the conclusions of this article will be made available by the authors, without undue reservation.

## Author contributions

SA: Writing – original draft. NT: Writing – review & editing. KA: Writing – review & editing. MC: Writing – review & editing. MN: Writing – review & editing. NS: Writing – review & editing. JP: Writing – original draft. VG: Writing – original draft. GT: Writing – original draft.

## Appendix A. Questionnaire

1. In which country do you practice? (Open).
2. Which is your profession (including in training)?
3. Please specify the type of hospital where you work.
4. Which type of PET/CT tomograph do you operate?
5. Do you perform PET/CT imaging with contrast enhancement CT (PET/ceCT) in your centre?
6. Which is the overall percentage of PET/ceCT scans of the whole number of PET/CT scans in your centre in the last year (approximately)?
7. Do the clinical indications (oncological versus non oncological) affect the use of PET/ceCT in your centre?
8. Does the PET radiopharmaceutical used ([^18^F]FDG or other PET radiopharmaceuticals) affect the use of PET/ceCT in your centre?
9. Who do report PET/ceCT in clinical routine in your centre (multiple answers are possible)?
10. Please list up to three main clinical indications of PET/ceCT in your centre (Open).
